# Risk factors for *Brucella* spp. and *Coxiella burnetii* infection among small ruminants in Eastern India

**DOI:** 10.1080/20008686.2020.1783091

**Published:** 2020-06-23

**Authors:** Eithne Leahy, Rajeswari Shome, Ram Pratim Deka, Swati Sahay, Delia Grace, Stella Mazeri, Johanna F. Lindahl

**Affiliations:** a Royal (Dick) School of Veterinary Studies, The University of Edinburgh, Edinburgh, Scotland, UK; b Department ofAgricultural Research, ICAR- National Institute of Veterinary Epidemiology and Disease Informatics (ICAR-NIVEDI), Bengaluru, India; c Department of Agricultural Research, International Livestock Research Institute, Guwahati, Assam, India; d Department ofAgricultural Research, International Livestock Research Institute (ILRI), Nairobi, Kenya; e International Livestock Research Institute, Southeast Asia Regional Office, Hanoi, Vietnam; f Zoonosis Science Centre, Uppsala University, Uppsala, Sweden; g Department of Clinical Sciences, Swedish University of Agricultural Sciences, Uppsala, Sweden

**Keywords:** Brucellosis, coxiellosis, Q-fever, seroprevalence, zoonoses, risk factors

## Abstract

Small ruminants are the main reservoirs for brucellosis and coxiellosis, two zoonotic diseases affecting livestock production, and posing a public health threat in India. Understanding disease prevalence and risk factors associated with small ruminant infection can help mitigate disease transmission.

We report a cross-sectional survey in the states of Assam and Odisha in Eastern India. We interviewed 244 farmers to assess knowledge, attitude and practices relevant to brucellosis and coxiellosis infection. Serum samples from 411 goats and 21 sheep were analysed using enzyme-linked immunosorbent assay and Rose-Bengal *Brucella* agglutination plate test. Higher *Brucella* and *Coxiella burnetii* seroprevalence were found in Odisha (22% and 11.5%, respectively) than Assam (9.8% and 1.6%, respectively), and certain districts in Odisha were at higher risk. No association was found between seropositive animals and clinical signs, a challenge when attempting to identify seropositive animals in the herd. None of the farmers interviewed were aware of brucellosis, its aetiology, clinical form, or zoonotic risk. This study acts as a first indication of the extent of these diseases among small ruminants in these Indian states, highlighting how farming practices are associated with increased risk of infection. More research is urgently needed to mitigate zoonoses transmission in this region.

## Introduction

Sheep and goats help support the livelihoods of millions of poor rural households in India [1]. An estimated 98% of small ruminants are owned by small, landless, and often illiterate farmers [2]. Prioritising small ruminant health is challenging due to the ad hoc approach taken to goat and sheep rearing [3] and the difficulty in estimating small ruminant disease costs [4]. Disease prevention and control in small ruminants are further hampered by inadequate veterinary services in rural areas [3,4]. However, given that many zoonotic pathogens of serious animal and public health concern in India have small ruminants as reservoirs [5,6], it is of paramount public health importance to understand zoonoses prevalence in small ruminants.

Brucellosis is endemic in India [7,8] and sheep and goats are a major source of infection [9,10]. A sharp increase in human brucellosis in recent years has been attributed to *Brucella melitensis* [11,12], which is the species usually responsible for small ruminant brucellosis [13]. To date, a limited number of small ruminant studies have been carried out in India to understand brucellosis seroprevalence [1,14–16], but other infectious agents have been overlooked [17].

One such agent is *Coxiella burnetii*, causing coxiellosis, or Q-fever as it is called when affecting humans, a widely distributed zoonotic disease of animal and public health concern [6,18,19]. *Coxiella burnetii* has multiple hosts and its transmission is also affected by environmental factors [18]. It is ranked among the top 13 global priority zoonoses [20]. Small ruminants have been identified as one of its primary reservoirs [18,21,22]. Despite its ubiquitous nature globally [21], human cases are underdiagnosed and underreported in India [23], and little is understood of its prevalence in Indian livestock [24].

Brucellosis and coxiellosis in small ruminants are both characterised by abortion during late pregnancy, stillbirths or the delivery of weak kids, thus causing severe reproductive losses [22,25]. Both pathogens cause chronic infection in the uterus and mammary glands of infected goats and sheep [26] and are both shed mostly in placental membranes and birth fluids, but also in milk and faeces [27]. Both cause fever and chronic disease in humans [1], are resistant in the environment, can spread as aerosols and may cause large-scale outbreaks due to their low infectious dose [27]. Coxiellosis can also be transmitted to livestock via tick bites [18,22]. Control of either pathogen is challenging due to the latent nature of infection; a normal parturition often follows an abortion, making culling of infectious animals within a herd complicated [11,22].

In India, animal husbandry is the second largest occupation in rural areas [28]. A One Health approach including transdisciplinary collaboration and participation of communities is necessary for successful interventions to mitigate human infection at the animal, environmental, human interface. Prevention of human brucellosis and Q-fever from a public health perspective is based on control of the disease in the small ruminant reservoir [29]; thus, understanding how farmers interact with their sheep and goats is vital. In this study, a twofold approach examined the seroprevalence of *Brucella* and *C. burnetii* in small ruminants in the northeastern Indian states of Assam and Odisha. These states were chosen due to the dearth of reports on small ruminant zoonoses [2,12,30]. A farmer knowledge, attitude, and practices (KAP) questionnaire was used to gain a deeper understanding into farming practices, rearing conditions, and contact between small ruminants and other species, all important risk factors identified for *C. burnetii* and *Brucella* infection [2,22]. Through an integrated approach, combining serology sampling with farmer interviews, this study created a deeper understanding of the epidemiology and risk factors for *Brucella* and *Coxiella* seropositivity, benefiting future intervention programmes and policy development in mitigating transmission risk in Eastern India.

## Materials and methods

### Sampling design

This cross-sectional study was conducted in the Indian states of Assam and Odisha from March to December 2017, using a multistage sampling technique for household sample selection in both states. The first stage was to select three districts each from the 33 districts in Assam and 30 districts in Odisha. District selection was guided by consultations with the Animal Husbandry and Veterinary Department officials of each state who have access to goat and sheep census numbers. Accordingly, Kamrup, Bangaigaon, and Sonitpur districts were selected in Assam, and Cuttack, Kendrapara, and Mayurbhanj districts selected in Odisha. Secondly, two community development blocks (CDBs) from each district, one urban and one rural were randomly selected. Thirdly, two villages were selected randomly from each CDB. A list of households with goats and sheep in each of these selected villages was created with the support of key informants, including the local non-governmental organisations (NGO) and village headsmen. From these lists, 10 households were selected randomly, however from two villages in Assam and Odisha, 11 households per village were included. One hundred and twenty-two households were thus selected in total from each state for the study. Random selection was done using the random number function in MS Excel.

### Ethics statement

Ethical permission for the study was granted by the Institutional Research Ethical Committee of the International Livestock Research Institute ILRI-IREC2017-39 as well as by the ethical board at Indian Council of Agricultural Research-National Institute of Veterinary Epidemiology and Disease Informatics (ICAR-NIVEDI).

### Data collection

The farmers were contacted before the study by key informants. Informed consent was obtained from all participants included in the study and farmers were compensated for their time. A pre-tested household questionnaire with closed questions was distributed by the veterinary scientists who conducted the fieldwork at the time of blood sampling and who spoke the local language. Two male interviewers worked in the state of Odisha and five male interviewers worked in the state of Assam. The questionnaire was piloted in the area before the start of the survey, and the interviewing personnel were trained in order to have a common methodology. Knowledge on zoonoses was assessed, with only brucellosis being named since animal health extension efforts have until now only ever focused on this disease.

### Serological sampling

To evaluate seroprevalence, the aim was to sample two randomly selected female small ruminants per farm, although sometimes farmers only had one animal, or only allowed sampling of one. Blood samples from 411 goats and 21 sheep were collected from the jugular vein into a sterile syringe, transferred to vacutainer tubes, allowed to clot, and stored using ice packs before being transported back to a local lab where the samples were frozen until shipment to ICAR-NIVEDI.

#### Coxiella burnetii

The 432 serum samples were tested for antibodies against *C. burnetii* inactivated phase I and II antigens using an indirect commercial ELISA test as per the manufacturer’s instructions [31]. Results were expressed as a percentage of the optical density (%OD) reading of the test sample calculated as %OD = 100 * (S-N)/(P-N), where S, N, and P are the values of the sample (S) and OD of negative (N) and positive (P) controls, respectively. Samples with %OD ≥37% were considered positive.

#### Brucella *spp.*

The 432 serum samples were tested for antibodies against *Brucella* spp. using an indirect, multi-species, commercial enzyme-linked immunosorbent assay (ELISA) kit as per the manufacturer’s instructions [32]. Results were expressed as a percentage of the optical density (%OD) as described above. Samples with %OD ≥ 30% were considered positive. According to the manufacturer, the test had 100% specificity and sensitivity for ovine sera [32].

The sera were also screened using a Rose-Bengal *Brucella* agglutination plate test (RBPT) following the standard procedure as described by 33. The plates were shaken for 4 min and any agglutination that appeared within this time was recorded as a positive reaction. The results were read by experienced technical staff.

### Statistical analysis

Data were entered into Microsoft Excel and analysed using ‘R’ statistical software [34]. Given scarcity of sheep numbers, for the purpose of this analysis, sheep and goats were grouped together and referred to using the term ‘small ruminant’. Initial univariable analyses were conducted using Chi2 testing to identify each potential risk variable firstly for *Brucella* and then for *Coxiella* seropositivity. Mixed-effects multivariable logistic regression models were built, starting with all independent variables with a p value <0.1 in the univariable analysis, using manual backward elimination of variables, with district and village as random effects for the farm level model and farms as a random effect for the animal level model. The Goodman and Kruskal’s tau measure were used to check for correlated pairs of variables using a correlation matrix prior to the multivariable analysis. The R lme4 package [35] was used to fit the mixed-effects multivariable logistic regression models as described above. The lowest Akaike’s Information Criteria (AIC) value was used as a measure of best model parsimony. Maps were created in ‘R’ with the R leaflet package [36].

## Results

In total 244 farms were visited, 122 farms per state, with one farmer questionnaire administered per farm. Of the 244 respondents of the farmer questionnaire, 133 were male and 111 were female. The average age of male farmers was 45.7 years and 39.4 years for females. A mean of five people lived in each farming household. Odisha had larger herd sizes compared to Assam; mean number of goats and sheep per farm in Odisha was 7.8 goats and 2 sheep compared to 4.7 goats and 0.2 sheep in Assam. Five farms reported to produce small ruminant milk, while most animals were kept for meat production.

**Figure 1.**
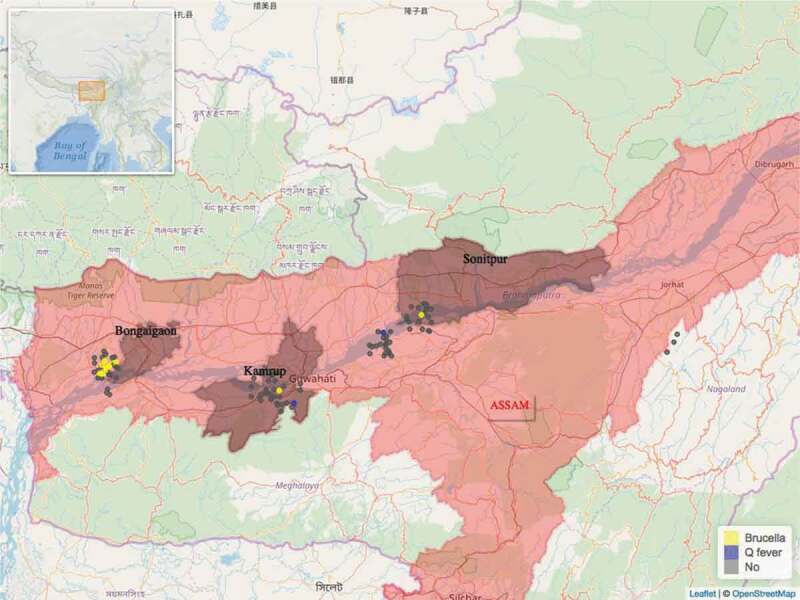
Distribution of the study farms within the state of Assam and the location of Assam within India (insert). Seropositive farms for *Brucella* spp. are in yellow and seropositive farms for *Coxiella burnetti* are in blue colour.

**Figure 2.**
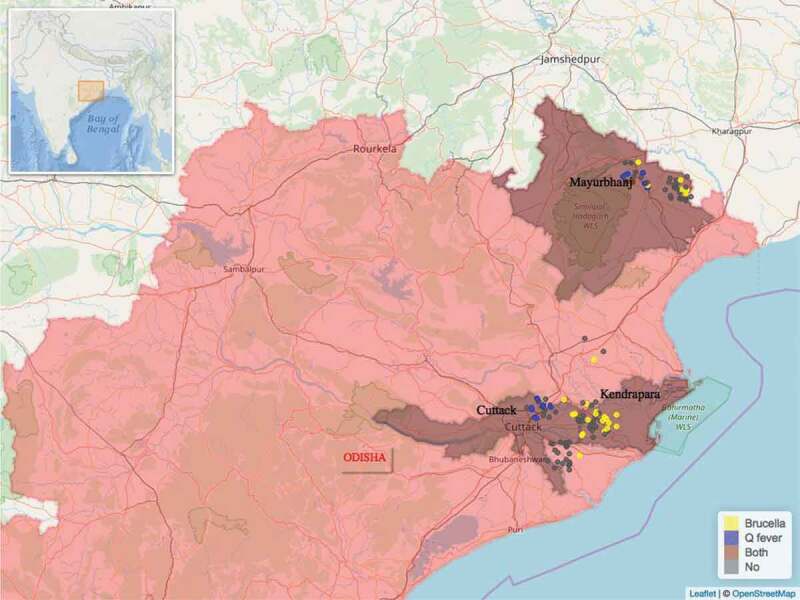
Distribution of the study farms within the state of Odisha and the location of Odisha within India (insert). Seropositive farms for *Brucella* spp. are in yellow colour and seropositive farms for *Coxiella burnetti* are in blue colour. Only one farm, in brown colour, had animals seropositive for both *Brucella* spp. and *Coxiella burnetti.*

In total 432 blood samples were collected from 411 goats and 21 sheep. All 21 sheep were of local indigenous breed. Three of the 411 goats were crossbreeds, all remaining 408 goats were of local indigenous breed. Of the 432 blood samples, 43 were found to be seropositive for *Brucella*, 20 were seropositive for *Coxiella* and 1 sample was seropositive for both pathogens. Of the 244 farms visited, 53 were seropositive, i.e. a farm where one or more seropositive animals were found. The distribution of the 53 seropositive and 191 seronegative farms found can be seen on the maps in Figures 1 and 2. Farm seroprevalence for *Brucella* in Odisha was 22% (95% CI 15.6%-30%) and 9.8% (95% CI 5.7%-16%) in Assam. Farm seroprevalence for *Coxiella* in Odisha was 11.5% (95% CI 7%-18%) and 1.6% (95% CI 0.5%-6%) in Assam.

### Analysis of factors associated with Brucella and Coxiella infection at the farm level

Univariable analysis identified Odisha state and certain districts as having higher *Brucella* and *Coxiella* farm seroprevalence. Odisha’s district of Kendrapara was associated with high *Brucella* seroprevalence risk while Cuttack was associated with high *Coxiella* seroprevalence risk. Small ruminants kept by men had higher *Brucella* seroprevalence risk. Farmer age and education level were not identified as risk factors. In terms of farm hygiene, cleanliness of farms, use of disinfectant, and farmers’ hand-washing habits were not associated with seropositivity risk. Results of the univariable analysis are shown in Table 1.

**Table 1.** Univariable analysis showing risk factors associated with *Brucella* and *Coxiella* farm seroprevalence (with 95% confidence interval (CI)).

Risk Factor	Levels	*Brucella* seropositivity (95% CI)	p-value*	*Coxiella* seropositivity (95% CI)	p-value*
State	Odisha Assam	22% (15.6%–30%) 9.8% (5.7%–16%)	p = 0.008	11.5% (7%–18%) 1.6% (0.5%–6%)	p < 0.001
District	Kendrapara Mayurbhanj Cuttack Bangaigaon Kamrup Sonitpur	43.9% (30%–59%) 19.5% (10%–34%) 2.5% (0.1%–13%) 25% (14%–40%) 2.5% (0.1%–13%) 2.4% (0.1%–12%)	p < 0.001	2.4% (0.1%–12%) 17% (8.5%–31%) 17.5% (8.7%–32%) 0% 2.5% (0.1%–12.8%) 2.4% (0.1% −12%)	p < 0.001
Gender	Male Female	22% (15.6%–30%) 9% (5%–15.8%)	p = 0.006	7.5% (3.7%–13.3%) 5.4% (2.0%–11.4%)	p = 0.69
Floor Type	Earthen Other	15% (11%–20%) 40% (17% −69%)	p = 0.03	6.8% (4%–11%)	p = 0.4
Vaccination Status	Vaccinated Unvaccinated	28% (18% −40%) 12% (8% – 18%)	p = 0.006	28% (18%–40%) 12% (8%–18%)	p = 0.4
Total herd size	< 5 small ruminants 5–10 small ruminants >10 small ruminants	19% (11%–31%) 13% (8%–20%) 21% (12%–35%)	p = 0.3	12% (6%–23%) 2% (1%–7%) 13% (6%–25%)	p = 0.006
Introduced new animals into herd	< 5 small ruminants 5–10 small ruminants >10 small ruminants	15% (11%–21%) 23% (10%–43%) 17% (5%–45%)	p = 0.658	5% (2%–8%) 9% (3%–28%) 33% (14%–61%)	p < 0.001
Small ruminants mixing with poultry	Yes No	11% (6.6% −18%) 22% (15% – 30%)	p = 0.024	6% (3%–11%)	p = 0.5
Small ruminants mixing with dogs	Yes No	11% (6.5% −18%) 21% (15% −29%)	p = 0.035	2% (0.5%–6%) 11% (7%–18%)	p = 0.003
Small ruminants mixing with cats	Yes No	11% (6.4% −18%) 20% (14% −28%)	p = 0.057	2% (0.5% – 6.4%) 10% (6.3% – 17%)	p = 0.007
Vet visiting farm	Vet visit No vet visit	12% (7% −19%) 20% (14 − 27%)	p = 0.238	1.8% (0.5% – 6.4%) 11% (6.4% −17%)	p = 0.023

*Chi-square test

In Odisha, 60 farms vaccinated small ruminants against peste des petits ruminants (PPR), foot and mouth disease (FMD) and haemorrhagic septicaemia (HS), while only one farm in Assam vaccinated small ruminants. Vaccinated farms were associated with increased brucellosis seroprevalence risk. Farms where small ruminant houses were of a floor type different to earthen had higher brucellosis risk. Larger herd size and increased introduction of new animals into a herd were risk factors for *Coxiella* farm prevalence.

No farm reported to have purchased sick or weak animals over the previous 12 months, only two farms in Assam reported an animal becoming sick after purchase, this was not significantly associated with any seroprevalence risk. Two farms reported culling of animals. No farmer knew of or used quarantine methods. No farmer possessed knowledge of brucellosis, its aetiology, clinical form, or zoonotic risk.

No association was found between placenta disposal on farms and seroprevalence or abortion cases. Of the 31 farms that reported cases of small ruminants aborting, 54.8% (95% CI 36.0%-72.7%) throw the placenta into an open field, 29.0% (95% CI 14.2%-48.0%) throw it in an open drain and 16.1% (95% CI 5.5%-33.7%) bury it. Most farmers, 86% (95% CI 82%-91%), lived adjacent to their small ruminant house, no association between farm seropositivity and proximity of small ruminant shed to residential homes was found.

Higher numbers of farms in Assam allowed small ruminants to mix with other animal sources of *Brucella* spp. and *C. burnetii*: poultry (119 farms in Assam, 9 farms in Odisha), cattle (121 farms in Assam, 50 in Odisha), dogs (117 farms in Assam, 2 in Odisha) cats (109 farms in Assam, 0 in Odisha) and pigs (23 farms in Assam, 0 in Odisha). In the univariable analysis poultry, dogs, and cats mixing with small ruminants was associated with lower *Brucella* and *C. burnetii* farm seroprevalence. No association was found between small ruminants mixing with cattle or pigs and farm seroprevalence. In Assam, 108 farms reported veterinary visits over the previous 12 months compared to 3 farms in Odisha. Veterinary visits to farms were associated with lower *Coxiella* seropositivity risk (Table 1).

Results of the multivariable analyses modelling for combined *Brucella* and *Coxiella* seropositivity at farm level are shown in Table 2. For both *Brucella* and *Coxiella* seroprevalence at the farm level, the most parsimonious model showed that state and mixing with poultry were significantly associated. If mixing with poultry was removed from this model, the AIC value increased with estimates for state-changing, thus highlighting it as a confounding variable. Farms in Odisha had far higher odds ratio (OR) of being seropositive for either pathogen than farms in Assam (OR = 17.4, 95% CI 1.5%-190%).

**Table 2.** Multivariable analyses of risk factors for combined seropositivity of *Coxiella* and *Brucella.*

Risk factors farm level seroprevalence	Odds Ratio	95% Confidence Interval
State	17.4	1.5–190
Mixing with poultry	3.6	0.5–24
Farm level random effects: Village District	Groups Variance 2.59 3.44 × 10^−9^	Std.Dev. 1.61 5.86 × 10^−5^

### Analysis of factors associated with Brucella and Coxiella infection at animal level

Results of the univariable analysis at the animal level are presented in Table 3. Out of 433 sampled small ruminants, 64 were seropositive for either or both bacteria. In Odisha 32 (14%, 95% CI 10–19%) were positive for *Brucella* spp. and 12 (6%, 95% CI 3.5%-10) in Assam, while 19 small ruminants were seropositive for *C. burnetii* in Odisha (8%, 95% CI 5–12%) and 2 (1%, 95% CI 0.3–4%) in Assam. Only one sheep in Odisha was seropositive for both pathogens (0.43%, 95% CI 0.022–2.4%). The ELISA kit for *Brucella* spp. yielded 43 positives results, higher than the RBPT method which gave one positive result. Few differences were found between risk factors already identified at farm level with those found at individual animal level; small ruminant shed floor type was no longer seen as a risk factor for *Brucella* seroprevalence and small ruminants grazing extensively full time were found to be associated with increased risk of *Coxiella* seroprevalence. History of abortions, number of pregnancies, age, and species were not associated with *Coxiella* seroprevalence. Older animals and sheep, more than goats, were identified as risk factors for *Brucella* seropositivity. No association was found between seropositive small ruminants and the presentation of clinical signs; abortion, mastitis, vaginal discharge, or lameness.

**Table 3.** Univariable analysis showing risk factors associated with *Brucella* and *Coxiella* seropositivity at animal level. Results shown as seropositivity (95% confidence interval (CI)) or mean (standard deviation (SD)).

Risk Factor	Levels	*Brucella* animal seropositivity (95% CI)	p-value*	*Coxiella* animal seropositivity (95% CI)	p-value*
State	Odisha Assam	14% (10%–19%) 6% (3.5%–10%)	p = 0.008	8% (5%–12%) 1% (0.3%–4%)	p < 0.001
District	Kendrapara Mayurbhanj Cuttack Bangaigaon Kamrup Sonitpur	29% (20%–40%) 11% (6%–19%) 1% (0.2%–7%) 14% (8%–24%) 2% (0.1%–9%) 1% (0.1%–8%)	p < 0.001	0% - 13% (8%–22%) 11% (5.5%–20%) 0% - 2% (0.1%–9%) 1% (0.1%–8%)	p < 0.001
Gender	Male Female	14% (10%–19%) 5% (3%–10%)	p = 0.003	5% (3%–9%) 4% (2%–8%)	p = 0.6
Vaccination Status	Vaccinated Unvaccinated	17% (11%–25%) 8% (5%–11%)	p = 0.004	3% (1%–7%) 6% (4%–9%)	p = 0.2
Species	Sheep Goat	36% (20 − 57%) 9% (6 − 12%)	p < 0.001	5% (0.23 − 22%) 5% (3 − 7%)	p = 0.9
Total herd size	< 5 small ruminants 5–10 small ruminants >10 small ruminants	0% 0% 10% (8%–14%)	p = 0.8	0%- 0%- 5% (3%–7%)	p < 0.001
Introduced new animals into herd	< 5 small ruminants 5–10 small ruminants >10 small ruminants	10% (7%–13%) 11% (5%–24%) 19%(8%–40%)	p = 0.4	4% (2%–6%) 5% (1%–15%) 24% (11%–45%)	p < 0.001
Rearing Systems	Part time grazing/stalled Full time grazing	8% (4%–15%) 11% (8%–15%)	p = 0.4	-7% (4%–9%)	p = 0.017
Small ruminants mixing with poultry	Yes No	11% (6.6% −18%) 22% (15% – 30%)	p = 0.024	5% (3–9%) 5% (3–9%)	p = 0.9
Small ruminants mixing with dogs	Yes No	11% (6.5% −18%) 21% (15% −29%)	p = 0.035	2% (0.5%–6%) 11% (7%–18%)	p = 0.003
Small ruminants mixing with cats	Yes No	11% (6.4% −18%) 20% (14% −28%)	p = 0.057	2% (0.5% – 6.4%) 10% (6.3% – 17%)	p = 0.007
Vet visiting farm	Vet visit No vet visit	7% (4%–12%) 13% (9%–17%)	p = 0.2	1% (0.3%–4%) 8% (5%–12%)	p = 0.007
Animal age	Seropositive Seronegative	Mean (SD), 3.4 (1.5) 2.9 (1.4)	p = 0.029 ^t^	mean (SD) 3 (1.4) 2.6 (1)	p = 0.3 ^t^

*Chi-square test t = t-test

In the multivariable model at animal level, the most parsimonious model for *Brucella* showed that district, gender, and mixing with poultry were significantly associated with seropositive animals (Table 4). Small ruminants living in Odisha’s Kendrapara district had 5.9 (95% CI 1%-34%) higher odds or being *Brucella* seropositive compared to Mayurbhanj or Cuttack district. Small ruminants mixing with poultry had 3.3 (95% CI 0.7–15) times higher odds of being at risk compared to those coming from herds who did not mix with poultry. Due to the low number of *Coxiella* seropositive small ruminants, the multivariable mixed model did not converge, so results are not shown here.

**Table 4.** Multivariable model for risk factors for *Brucella* seropositivity at animal level.

Risk factors *Brucella* spp.	Odds Ratio	95% Confidence Interval
District (Kendrapara)	5.9	1–34
Mixing with poultry	3.3	0.7–15
Gender	0.4	0.2–1

## Discussion

This study is the first of its kind to look at the spatial distribution and risk factors for both *Brucella* and *Coxiella* seroprevalence in small ruminants in Assam and Odisha in Eastern India. In Assam high *Coxiella* seroprevalence has already been reported in cattle and identified as a public health risk [37], but no reports on coxiellosis in small ruminants exist [30]. In Odisha, *Coxiella* seroprevalence in goats has been reported at 10.6% [2], higher than the 8% (95% CI 5–12%) we reported. For *Brucella* spp., our study found a seroprevalence of 14% in Odisha (95% CI 10–19%), and 6% in Assam (95% CI 4–10%), higher than observations previously made of 5% seroprevalence among sheep and goats in Odisha [15], and a 2% seroprevalence recorded in goats in Assam [38]. Previous studies have however shown particularly high prevalence (70% herd prevalence) in cows in peri-urban Guwahati, the capital of Assam [39].

Our study found 43 seropositive animals for *Brucella* spp. using the ELISA method compared to one positive animal found using RBPT. This result supports findings from previous Indian studies that report a higher diagnostic sensitivity of the ELISA compared to RBPT method [40–42]. The RBPT method serves an indirect function, however. Standard RBPT favours *B. abortus* with *B. melitensis*-infected small ruminants showing negative results [43]. Therefore, one positive result using RBPT compared to 43 positives using ELISA indicates that the small ruminants sampled are likely to be infected with *B. melitensis* rather than *B. abortus*, an important public health finding given that *B. melitensis* is more pathogenic to humans [9,12].

If brucellosis serology testing alone had been carried out in this study and coxiellosis neglected, our combined farm seroprevalence would have decreased from 15% to 10%, a total of 21 infected small ruminants (19 in Odisha and 2 in Assam) would have been undetected. These 21 animals highlight the benefits of extending beyond single serology screening approaches, gaining greater insights into zoonoses prevalence.

To understand risk factors associated with brucellosis and coxiellosis infection, serology alone can be misleading; a significant proportion of animals that shed *C. burnetii* or *Brucella* spp. are not seropositive, furthermore animals can be seropositive and not shed the pathogen [21,22,27]. In a developing world context, studies which have focused solely on serology have failed to give sufficient insight into sustainable control options [44]. Therefore in this study, a farmer questionnaire was used to deepen our understanding of animal-human-environmental contact patterns, necessary information to reduce zoonotic transmission risks [45].

In terms of knowledge about brucellosis, transmission pathways, or control measures, none of the 244 farm respondents possessed any information, this concurs with other research showing a strong lack of knowledge among Indian livestock keepers on zoonotic disease and highlights the urgent need for intervention [6,46]. Our study shows the proximity within which small ruminants and their keepers live, 86% of respondents in this study live adjacent to their small ruminant house. Increased brucellosis and coxiellosis transmission to humans and other animals occurs through exposure to placenta membranes and birth fluids from infected small ruminants [22,29,47], and so we investigated methods of placenta disposal on farms. We found more than half of the farmers throw the placenta from aborted small ruminants into an open field, or in an open drain, and only 16% bury it. While no association was found between placenta disposal and seropositive farms or farms reporting abortions, inappropriate disposal of hazardous farm waste material must be discouraged to reduce environmental contamination and intra- and inter-species transmission.

No farmer practiced quarantine in the study. Increased herd numbers, increased introduction of new small ruminants into herds, and full-time extensively grazed small ruminants were associated with higher *Coxiella* seroprevalence risk, all highlighting the opportunistic, infectious nature of this pathogen when herd density increases [18]. Increased farmer knowledge is needed regarding the implementation of biosecurity measures. In addition, the risks and cost-effectiveness of vaccination interventions, where the mixing of herds increases *Brucella* transmission [48] and close animal-human contact occurs [49], require further investigation given our findings of increased *Brucella* seroprevalence among vaccinated small ruminants.

Inapparent or ‘silent’ clinical signs of brucellosis and coxiellosis in small ruminants complicates their clinical diagnosis [21,29]. We found no association between *Brucella* or *Coxiella* seropositive small ruminants with clinical signs; abortion, mastitis, lameness, or vaginal discharge. Farms that reported abortion did not report infertility and only two farms reported to have culled animals, suggesting a poor perception level among farmers regarding small ruminant production parameters. The increased risk of *Brucella* seroprevalence in older small ruminants, as seen in our study, could help identify animals for culling. However, engaging with farmers for future disease control plans will be challenging given their current lack of zoonoses knowledge and the lack of tangible disease manifestation in their herds.

The role of poultry in disseminating brucellosis to man and other animals is well reported [50–52], with dogs and cats also acting as mechanical disseminators of brucellosis and coxiellosis [22,25]. The apparent protective factor of small ruminants mixing with poultry, dogs, and cats associated with lower seroprevalence found in our univariable analysis is misleading. Mixing with these species is highly correlated with the state of Assam, where infections were less common. Increased seroprevalence risk when small ruminants are in contact with poultry did come out in the multivariable model. The ecology of farms in Assam and Odisha differs greatly, future epidemiological investigations should compare similar farming systems to truly understand the risks associated multi-species mixing on farms. Sheep and goats can infect cattle with *B. melitensis* [7,53], and given the public health implications of this, brucellosis transmission risks posed by small ruminants to large ruminant’s merits further investigation. To mitigate risk at the animal-human-environmental interface, control measures for all livestock species with shared pastures are recommended [29].

In India, field veterinarians have been reported to lack knowledge on zoonoses transmission risks [54]. Our study showed farms which had not received veterinary visits were associated with a higher seroprevalence risk. However, like the mixing with other species variables, a disproportionate number, 103, farms in Assam where seroprevalence is lower, compared to 3 farms in Odisha, where seroprevalence is higher, received veterinary visits suggestive of a strong correlation with state. A limitation to the questionnaire design was that the motive for the veterinary visit to farms was not recorded, this would have been of interest in furthering our understanding of animal health priorities among small ruminant keepers as well as gaining a better insight into the role of veterinarians in small ruminant medicine.

Future studies may yield more insight into risk factors if more farming systems in similar settings were compared. Nevertheless, our study contributes to the current dearth of literature on coxiellosis and brucellosis in small ruminants in India. It identifies the state of Odisha and certain districts within Odisha as higher risk for both *Brucella* and *Coxeilla* farm seropositivity. Human health services in these areas must be made aware of such prevalence in the small ruminant reservoir and the potential human health risk. The study strongly identifies small ruminant farmers as target groups for urgent zoonosis educational intervention, with special emphasis on male farmers given how gender was associated with higher *Brucella* seroprevalence risk. The study also highlights the need for increased understanding on veterinary involvement in zoonotic disease mitigation. The inherent farm, animal and biosecurity-related factors identified as risks for seroprevalence in this study will need to be addressed if the complex landscape of interacting agents contributing to disease emergence [55], in this case brucellosis and coxiellosis, is to be understood. Further epidemiological investigation, to fully understand the transmission pathways on farms of both pathogens, is urgently needed if zoonotic risk in Eastern India is to be mitigated.
